# Doping of Mg on ZnO Nanorods Demonstrated Improved Photocatalytic Degradation and Antimicrobial Potential with Molecular Docking Analysis

**DOI:** 10.1186/s11671-021-03537-8

**Published:** 2021-05-01

**Authors:** Muhammad Ikram, Sidra Aslam, Ali Haider, Sadia Naz, Anwar Ul-Hamid, Anum Shahzadi, Mujtaba Ikram, Junaid Haider, Syed Ossama Ali Ahmad, Alvina Rafiq Butt

**Affiliations:** 1 Solar Cell Applications Research Lab, Department of Physics, Government College University, Lahore, Punjab 54000 Pakistan; 2 Physics Department, Lahore Garrison University, Lahore, Punjab 54000 Pakistan; 3 Department of Clinical Medicine and Surgery, University of Veterinary and Animal Sciences, Lahore, Punjab 54000 Pakistan; 4 Tianjin Institute of Industrial Biotechnology, Chinese Academy of Sciences, Tianjin, 300308 China; 5 Core Research Facilities, King Fahd University of Petroleum & Minerals, Dhahran, 31261 Saudi Arabia; 6 University College of Pharmacy, University of the Punjab, Lahore, 54000 Pakistan; 7 Institute of Chemical Engineering and Technology (ICET), University of the Punjab, Lahore, 54000 Pakistan

**Keywords:** ZnO, Co-precipitation, Nanorods, Photocatalysis, Sonocatalysis, Sonophotocatalysis

## Abstract

Various concentrations of Mg-doped ZnO nanorods (NRs) were prepared using co-precipitation technique. The objective of this study was to improve the photocatalytic properties of ZnO. The effect of Mg doping on the structure, phase constitution, functional groups presence, optical properties, elemental composition, surface morphology and microstructure of ZnO was evaluated with XRD, FTIR, UV–Vis spectrophotometer, EDS, and HR-TEM, respectively. Optical absorption spectra obtained from the prepared samples showed evidence of blueshift upon doping. XRD results revealed hexagonal wurtzite phase of nanocomposite with a gradual decrease in crystallite size with Mg addition. PL spectroscopy showed trapping efficiency and migration of charge carriers with electron–hole recombination behavior, while HR-TEM estimated interlayer d-spacing. The presence of chemical bonding, vibration modes and functional groups at the interface of ZnO was revealed by FTIR and Raman spectra. In this study, photocatalytic, sonocatalytic and sonophotocatalytic performance of prepared NRs was systematically investigated by degrading a mixture of methylene blue and ciprofloxacin (MBCF). Experimental results suggested that improved degradation performance was shown by Mg-doped ZnO NRs. We believe that the product synthesized in this study will prove to be a beneficial and promising photocatalyst for wastewater treatment. Conclusively, Mg-doped ZnO exhibited substantial (*p* < 0.05) efficacy against gram-negative (G-ve) as compared to gram-positive (G+ve) bacteria. In silico molecular docking studies of Mg-doped ZnO NRs against DHFR (binding score: − 7.518 kcal/mol), DHPS (binding score: − 6.973 kcal/mol) and FabH (− 6.548 kcal/mol) of *E. coli* predicted inhibition of given enzymes as possible mechanism behind their bactericidal activity.

## Introduction

Organic pollutant effluents in water and infectious bacterial contaminants in food items are becoming leading challenges that need to be overcome in order to sustain a healthy environment in our surroundings [1, 2]. As an example, infections caused by *Shigellaflexneri* bacteria claim around 1.5 million deaths by the year due to food and drinks contamination [3]. Toxic and carcinogenic agents present within dyes released into aquatic environment pose serious risks to the environment and public health [4]. These dyes also affect photosynthetic activity of aquatic lifeforms such as cyanobacteria and algae that serve to decrease the transparency of freshwater [5].

Innumerable experimental studies have been carried out to develop physical, biological and chemical methods as well as new technologies for dyes removal from wastewater. Until now, physical methods including ultra-filtration membrane, adsorption and precipitation [6] and biological approaches were studied. Furthermore, biodegradation procedures have been used for soluble organic matter degradation to eradicate bacteria that exist in discharges, while chemical methods are comprised of photochemical decolorization, chlorination and ozonation [7]. Conventional methods of wastewater treatment, including chemical precipitation, adsorption, coagulation and separation, are not suitable techniques since they require the transfer of dyes from one point to another and cause secondary contamination [8]. Therefore, researchers are looking for eco-friendly treatment technologies that involve direct degradation of organic pollutants into harmless compounds [9].

Recently, photocatalytic and sonocatalytic advanced oxidation processes (AOPs), in the presence of semiconductor nanoparticles (NPs), have gained much consideration due to their chemical stability, cost-effectiveness and non-toxicity [10–12]. Photocatalysis is an improved oxidation method, which involves charge carriers generation in semiconductor photocatalyst upon light irradiation. Photo-generated charge carriers participate in redox reactions and remove pollutants from water [13, 14]. Several studies have shown that OH^·^ radical species get accumulated on photocatalyst surface during photochemical reactions and lead to degradation of various organic dyes. Nowadays, as a consequence of an increase in the production of OH^·^ ions, the synergism of photocatalytic (PCA) and ultrasonic irradiation, so-called sonophotocatalysis (SPCA), appears to increase nanocatalyst degradation efficiency. In fact, SPCA has shown to have a beneficial impact on the degradation rate of chemical compounds in water that are toxic, dangerous and poisonous [15]. Currently, use of metal oxide NPs for the treatment of polluted water, due to their cost benefit, environmental friendliness, stability and recyclability, has attracted researchers' interest [16, 17]. Besides, broad bandgap inorganic semiconductors such as TiO_2_, WO_3,_ ZrO_2_ and ZnO have proven to be successful in light-induced catalytic redox processes to degrade dye [18, 19]. ZnO, a well-known wide bandgap (Eg=3.37eV) semiconductor, exhibits extraordinary potential owing to active surface defect sites in PCA applications, outstanding physiochemical stability, high oxidation–reduction potential, large binding energy of excitons (∼​60 meV), in addition to being inexpensive and toxic-free [20–23]. Among various metals, magnesium (Mg) is the most fascinating dopant to synthesize optical Eg-engineered ZnO nanomaterials. Substitution of Mg in ZnO is favored because of the following factors; (i) lattice constants invariant, (ii) ionic radii are very close (Mg^+2^= 0.72 Å and Zn^+2^= 0.74 Å), (iii) high solubility of Mg in ZnO, (iv) doped ZnO provides an increase in Eg and UV–Vis luminescence intensity, which is useful for optoelectronic applications. Moreover, Mg-doped ZnO can serve as an effective photocatalyst for dye degradation and encouraging antibacterial agent as a result of its broad optical band gap [23].

In this research work, co-precipitation route was adopted to synthesize efficient Mg-doped ZnO nanocomposites for catalytic and bactericidal activities. The prepared samples were characterized through XRD, HR-TEM, EDS, FTIR, UV–Vis and Raman spectroscopy for detailed analysis. Catalytic activity of prepared samples was studied for degradation of a mixture of methylene blue and ciprofloxacin (MBCF), while anti-bacterial activity was tested against G +ve and G -ve bacteria. In addition, molecular docking studies were performed against dihydrofolate reductase (DHFR) and dihydropteroate synthase (DHPS) of folate biosynthetic pathway and *β*-ketoacyl-acyl carrier protein synthase III (FabH) of fatty acid biosynthetic pathway.

## Methods

The current study was aimed to improved photocatalytic degradation and antimicrobial potential with molecular docking analysis of Mg-doped ZnO nanorods.

### Materials

Zinc nitrate tetrahydrate (Zn(NO_3_).4H_2_O, 99.0%), magnesium chloride hexahydrate (MgCl_2._6H_2_O, 99.0 %) and sodium hydroxide (NaOH, 99.0 %) were received from Sigma-Aldrich.

### Synthesis of Mg-Doped Zinc Oxide (ZnO)

**Fig. 1:**
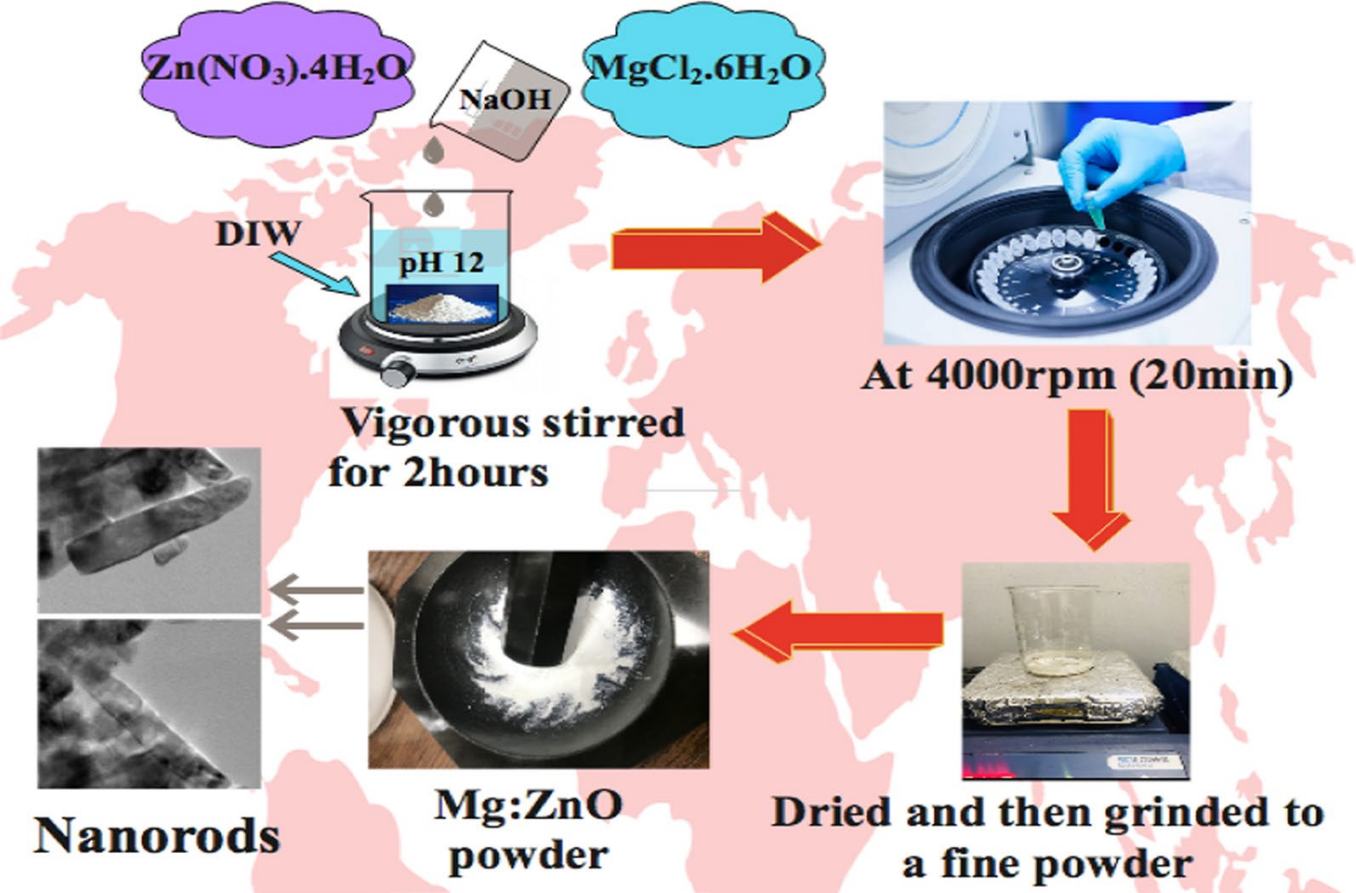
Schematic illustration of the synthesis stratagem of Mg-doped ZnO nanorods

Various concentrations of Mg-doped into a fixed amount of ZnO nanomaterials were synthesized with co-precipitation method. 0.5 M of Zn(NO_3_).4H_2_O solution was used as Zn precursor, and the desired amount (2, 4, 6 and 8 wt %) of dopant was added by pouring MgCl_2_ into the solution. Prepared solutions were stirred in deionized water (DI water) for 90 min at 80 °C, while pH was maintained around 12 by slowly adding NaOH (0.1 M) in stirred solution. Obtained precipitates were centrifuged at 4000 rpm (20 min), dried at 100 °C for 24 hours, and then ground to obtain fine powder (Fig. 1).

### Materials’ Characterization

In order to identify the phase constitution and structure of products, the PANanalytical X-pert PRO x-ray diffractometer-XRD equipped with CuK alpha-radiation (λ = 1.541874 Å) was operated in the 2θ° range (20°–80°). Existence of functional groups using the PerkinElmer spectrometer was verified via FTIR. With the UV–Vis spectrophotometer, optical properties were observed (Genesys 10S spectrophotometer). To acquire photoluminescence (PL) emission spectra in the 300–500 nm band, the JASCO FP-8200 spectrofluorometer was employed. With energy-dispersive X-ray spectroscopy (EDS) using INCA EDS software, elemental composition has been estimated. The scanning electron microscope (SEM model JEOL JSM 6460LV) and high-resolution transmission electron microscope (HR-TEM model JEOL JEM 2100F) were used to determine the morphology and microstructure of the synthesized samples.

### Photocatalytic, Sonocatalytic and Sonophotocatalytic Activity

Sonocatalytic (SCA) and sonophotocatalytic activity (SPCA) of the MBCF degradation was tested in ultrasonic baths for ZnO and Mg:ZnO catalysts operating at a fixed frequency ~ 35 kHz. Similarly, under visible light irradiation, photocatalytic degradation has been tested for ZnO and Mg:ZnO nanocatalysts against MBCF. In each experiment, in 50 mL model dye, photocatalyst (10 mg) was suspended and the solution was placed in dark for 10–15 min to achieve the adsorption–desorption equilibrium. Visible-light (photocatalysis—PCA), ultra-sonicator (sonocatalysis—SCA) and combined visible light irradiation with ultrasonication (sonophotocatalysis—SPCA) were methodically placed under suspended solutions. The 3 mL suspension was collected during exposure for absorption analysis at regular time intervals. Resulting dye constituent was observed by determining the difference in the λ max = 670 nm of MBCF. Blue color solution faded over time due to MBCF degradation in the presence of nanocatalysts. Finally, the degree of degradation (Ct/Co), where Ct is temporal dye concentration and Co is initial dye concentration, was evaluated. Percentage degradation for each sample was also calculated using equation, % Degradation = $\frac{{\left( {Co - Ct} \right)}}{Co}$ × 100.

### Isolation and Identification of *S. aureus* and *E. coli*

Dairy (bovine) milk samples tested with surf field mastitis were collected from different farms. The incubated samples (grown on 5% sheep blood agar) were streaked with MSA (Mannitol salt agar) and MA (MacConkey agar) for G+ve *S. aureus* and G-ve *E. coli*, respectively (pH ~ 7). Characteristic colonies were identified via biochemical (catalase and coagulase test) and morphological analysis (Gram staining).

### Antibacterial Activity

Bactericidal performance of synthesized NRs was examined on G-ve and G+ve bacterial strains employing agar well diffusion approach by swabbing 1.5 × 108 CFU/mL of *S.* *aureus* and *E. coli*isolates with MSA and MA, respectively. Wells with a diameter of 6 mm were formed using a sterile cork borer on swabbed MSA and MA petri dishes. In comparison with negative control (DI water) and positive control (ciprofloxacin), different concentrations of Mg:ZnO NRs (0.5 mg/50 μl) and (1.0 mg/50 μl) were used. The dose-contained petri dishes were incubated (37 °C) overnight, and anti-bacterial performance of NRs was recorded by measuring inhibition zones diameter with Vernier caliper. By means of one-way variance analysis (ANOVA) using SPSS 20, statistically measured efficacy in terms of inhibition zones was considered significant.

### Molecular Docking Studies

In silico molecular docking studies being an effective approach for identification of key structural feature behind antibacterial activity of doped ZnO NRs have been employed for prediction of their possible mechanism. The key enzymes of folate biosynthetic pathway, namely dihydrofolate reductase (DHFR) and dihydropteroate synthase (DHPS) alongside *β*-ketoacyl-acyl carrier protein synthase III (FabH) enzyme of fatty acid biosynthetic pathway, have been reported as an attractive target for antibiotics discovery. The 3D-structural characteristics of selected enzymes were retrieved from Protein Data Bank and prepared using protein preparation tool for docking of Mg-doped ZnO NRs inside active site.

The accession code for selected targets was as: 2ANQ (DHFR_E.coli_) [24], 5U0V (DHPS_E.coli_) [25] and 4Z8D (FabH_E.coli_) [26]. Molecular docking studies were performed using ICM Molsoft (Molsoft L.L.C., La Jolla, CA) software [27] where protein structures were optimized through energy minimization tool. The water molecules from crystal structure alongside co-crystallized ligand were removed followed by the addition of polar H-atoms for protein structure preparation, and grid box was used to identify active pocket. Finally, best docked complexes were selected for binding interaction analysis to observe key amino acids involved in ligand binding. Discovery studio visualizer and Pymol were used for analysis of docked complexes.

## Results and Discussion

**Fig. 2:**
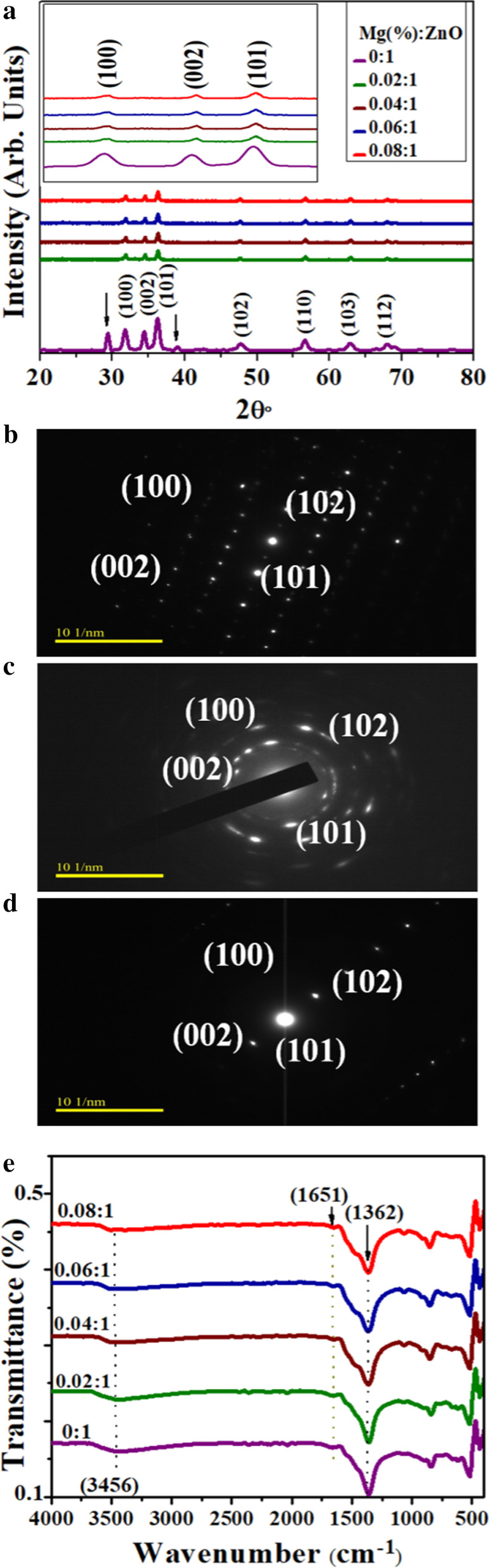
**a** XRD pattern of Mg-doped ZnO, **b–d** SAED pattern of ZnO, 4%, 8% of Mg-doped ZnO and **e** FTIR spectra, respectively

Structural properties and phase constitution of dopant-free and doped ZnO were assessed by using x-ray diffraction (Fig. 2a). Observed peaks at 31.7°, 34.5°, 36.3°, 47.5°, 56.6°, 62.9° and 68.0° can be assigned to diffractions planes (100), (002), (101), (102), (110), (103) and (112) that confirmed ZnO has hexagonal structure (JCPDS No. 361451) with space group P63mc. Two peaks reflect the impurities of compounds comprising zinc-carboxyl (marked by black arrows). These zinc-carboxyl traces might have appeared due to the reaction of Zn precursor with other reactants during synthesis [28]. The crystallite size of ZnO was 26 nm estimated using the Scherrer formula, which reduced gradually down to 23 nm with an increasing amount of dopant (at 8 wt%). To ensure successful addition of ‘Mg’ into host lattice, three prominent peak positions (100), (002) and (101) planes were tracked [23]. It is believed that ZnO peaks shift toward high 2θ upon doping with Mg, while several studies report a gradual reduction in crystallite size with the addition of dopants such as Mg, Fe and Al to ZnO [29, 30]. Several factors, including compression stress caused by the difference in the Zn ionic radii and dopant ion, crystal growth obstruction and/or defect generation in the crystals upon doping could suppress ZnO growth. Peaks shift observed for doped ZnO NRs could be attributed to Mg ions that substituted Zn ions due to ionic radii difference between Mg^2+^ (0.57 Å) and Zn^2+^ (0.60 Å) [23, 29].

SAED patterns of doped ZnO displayed bright spots due to electron diffraction. Each spot originated from a set of parallel planes found within the synthesized product crystal structure that affected the Bragg diffraction condition. Miller indices have been allocated accordingly as seen in Fig. 2b–d. Patterns have been indexed with planes (002), (100), (101) and (102) connected to hexagonal structure of ZnO with electron beam projecting along [101] zone axis [31]. In general, ZnO anisotropic direction of growth is determined by both interfacial free energy and water dissolution potential. The relative velocity of different planes growth also controls growth. The growth is also controlled by relative growth velocity of different planes [32].

FTIR analysis was performed to investigate the presence of functional groups, surface chemistry and modes of vibrations for chemical bonds existing in the samples (Fig. 2e). Bands from 400 to 560 cm^-1^ are designated to stretching of Zn–O–Zn vibrational modes that have confirmed the ZnO formation. Low-frequency/fingerprint region bands were ascribed to M–O translational vibrations (590, 670 cm^−1^) and O–M–O (430 cm^−1^) [33]. With increasing concentration of Mg, no significant change was observed in the absorption band of Zn-O and intensity. Band at 1651 cm^-1^ corresponds to symmetric C=O stretching mode that is highly intensified with increasing loading percentage of magnesium nitrate, while ~ 1362 cm^-1^ band corresponds to asymmetric C–O stretching mode. Carbon from starting materials could have been incorporated into NRs inadvertently, whereas appearance of broad transmission band at 3456 cm^-1^ corresponds to O–H stretching of surface adsorbed water molecules [34].

**Fig. 3:**
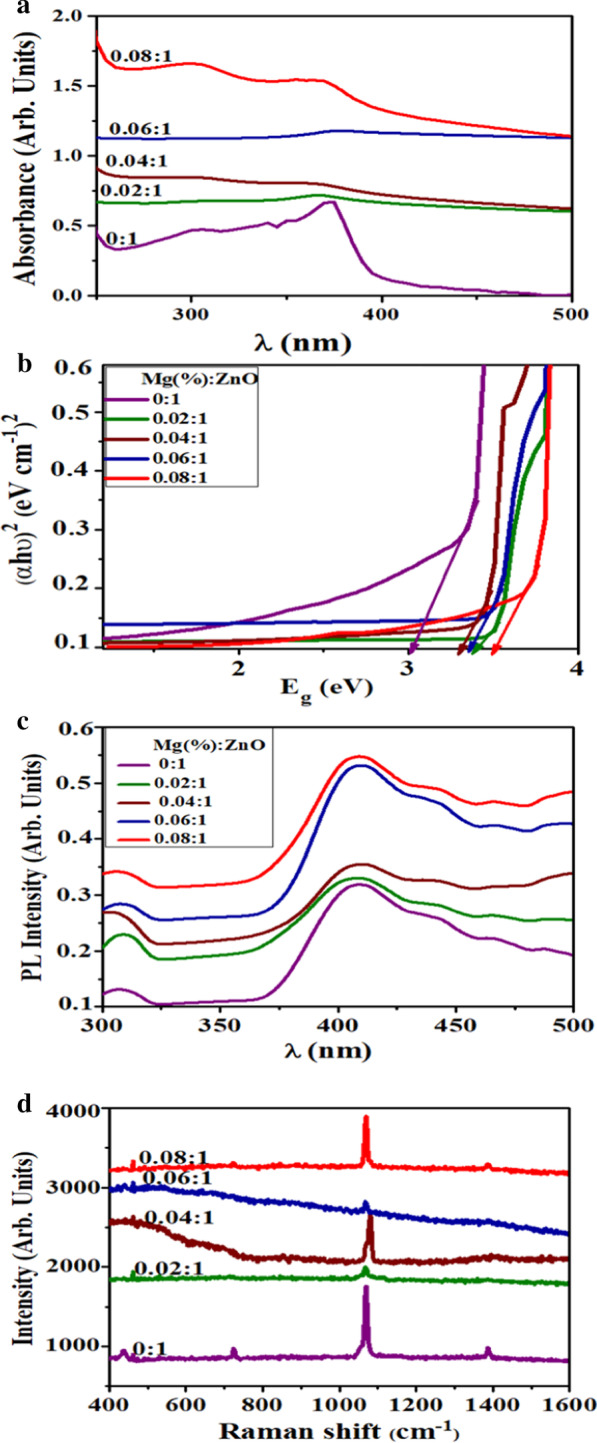
**a** Absorption spectra of samples of Mg-doped ZnO, **b** Tauc plot, **c** PL spectra and **d** Raman spectra of Mg-doped ZnO nanorods

In order to check the change in absorption behavior upon doping, UV–Vis spectrometry was deployed for doped and undoped samples. UV–Vis absorption spectra of synthesized NRs were recorded in the 250 to 600 nm range as a function of wavelength (Fig. 3a). Samples showed a maximum absorption around 370–395 nm, with a shift in absorption edge toward lower wavelength upon different doping concentrations. This increase in absorption and shift upon doping is manifested to oxygen deficiency, particle size effect and grain structure defects [35]. Extracted values from Fig. 3a were used to calculate optical band gap (Eg) of ZnO (using Tauc plot), which increased from 3.32 to 3.72 eV upon Mg doping (Fig. 3b) [36, 37]. This blueshift in Eg can be endorsed to the Burstein–Moss effect phenomenon. In the metal oxide method, particle size reduction is reported to result in a blueshift of the band gap due to the quantum confinement effect (QCE). However, QCE is not the only reason; doping may also affect local symmetry and generate lattice defect centers that change the structure of the band and induce significant shifts in optical properties [38]. As described earlier in XRD analysis, Mg doped into ZnO generates oxygen vacancies in host crystal, which act as donors in the system and behave as positively charged ions by releasing electrons to CB. As the concentration of the electron carriers exceeds the density of states in CB, the level of Fermi energy is pushed into the CB. Zn^2+^ substitute into Mg^2+^ leads to the increment in electron concentration and oxygen vacancy because of ionic radii and electronegativity difference of both materials, and thus, increase of carrier density leads the way to lifting of Fermi level to the degenerate semiconductor CB as ZnO is one of the most degenerate semiconductors. Due to this action, Fermi level as well as its position relies upon concentration of free electrons and excitation of electrons from VB to Fermi level, resulting in the increment of density of free electron and band gap widening [39]. This Burstein–Moss shift contributes to the observed Eg widening of Mg-doped ZnO NRs.

PL analysis is a valuable tool to get better information regarding impurities, transitions and dopants by studying emission spectra. Quantum size effects affect the physical properties of semiconductor materials at nanoscale, like ZnO changed its optical behavior by increase in quantum confinement observed from PL [40]. PL spectra of various concentrations of Mg incorporated into ZnO were measured with excitation λ~325 nm at room temperature (Fig. 3c). For undoped and doped ZnO, a broad deep level and near band emissions were detected. All samples showed an emission peak in the UV region, which is ascribed to exciton recombination. Peaks observed in the visible region appear due to defect states (donor), such as O_2_ vacancies-V_o_, Zn interstitials-Zn_i_, defect states (acceptor) from zinc vacancies-V_z_ and oxygen interstitials-O_i_ [39]. The peaks intensity ratio in the UV and visible region is mostly affected by crystal quality of doped materials, as the defects density decreases with crystallinity amplification. Samples demonstrated emission peaks around 408 nm ascribing to near-band edge-NBE transition of ZnO [39]. The peaks found at 408, 442, 467, 488 nm lead to blue emission and attributable to Zn interstitials have a major violet emission at 408 nm. Weak emissions observed at 442, 467 and 488 nm are indorsed to donor–acceptor (D/A) pair recombination that involves different defect levels in samples. Emission at 488 nm is due to electrons in singly ionized O_2_ vacancy with photo-excited holes in the VB [41]. The intensity of broad deep level emission increased upon doping, while NBE emission peaks were transferred to a higher energy region. This blue shift of NBE emission could be interpreted on the basis of Burstein–Moss effect. ZnO is an n-type material, and upon heavy doping, its Fermi level shifts inside conduction band. Thus, absorption must display blue shift as proposed by Burstein; filled regions would block optical or thermal excitations [42]. Increase in PL intensity was observed for doped samples implying reduced electron transfer efficiency.

Raman scattering is a sensitive and nondestructive technique to look into microstructure and analyze properties related to vibrational states of nanomaterials. Wurtzite zinc oxide with primitive cell containing two formula units is placed in C6ν space group. Optical phonons present at primitive cell in reciprocal space are justified from irreducible relation: Гopt = 1A_1_+2B_1_+ E_1_+2E_2_ where B_1_ represents Raman silent modes, while A_1_ and E_1_ are polar modes (long-range Coulomb forces), which are split into longitudinal optical (LO) and transverse optical (TO) phonons. Furthermore, a double-frequency phonon mode E_2_ (nonpolar), having E_2_ (low) and E_2_ (high), corresponded to Zn sub-lattice and O_2_ atoms [43]. In Raman spectra, the peaks that shift toward higher and lower wavenumber are dependent on varying bond lengths between molecules. Increase in bond length governs shift toward low wavenumber and vice versa. No further high-order peaks have been observed above 1300 cm^-1^(Fig. 3d). Dominant peak observed at ~ 1069 cm^-1^ represents E_2_H (characteristic) mode of hexagonal ZnO [44]. Moreover, three minor peaks were also observed around 436, 723 and 1386 cm^−1^ that originated due to high florescent background. Moreover, Raman spectrum of 8 wt% doped ZnO was blueshifted, which is attributed to substitution of Mg^2+^ with Zn^2+^ in ZnO lattice that is believed to play a role in lattice dynamics [45]. Usually, Raman peak shifts occur for three reasons: phonon confinement effects, lattice strain and oxygen vacancies. Acquired spectra from XRD and Raman spectroscopy confirmed that wurtzite-ZnO structure is unaffected by Mg incorporation; however, the quality of crystal is reduced significantly.

**Fig. 4:**
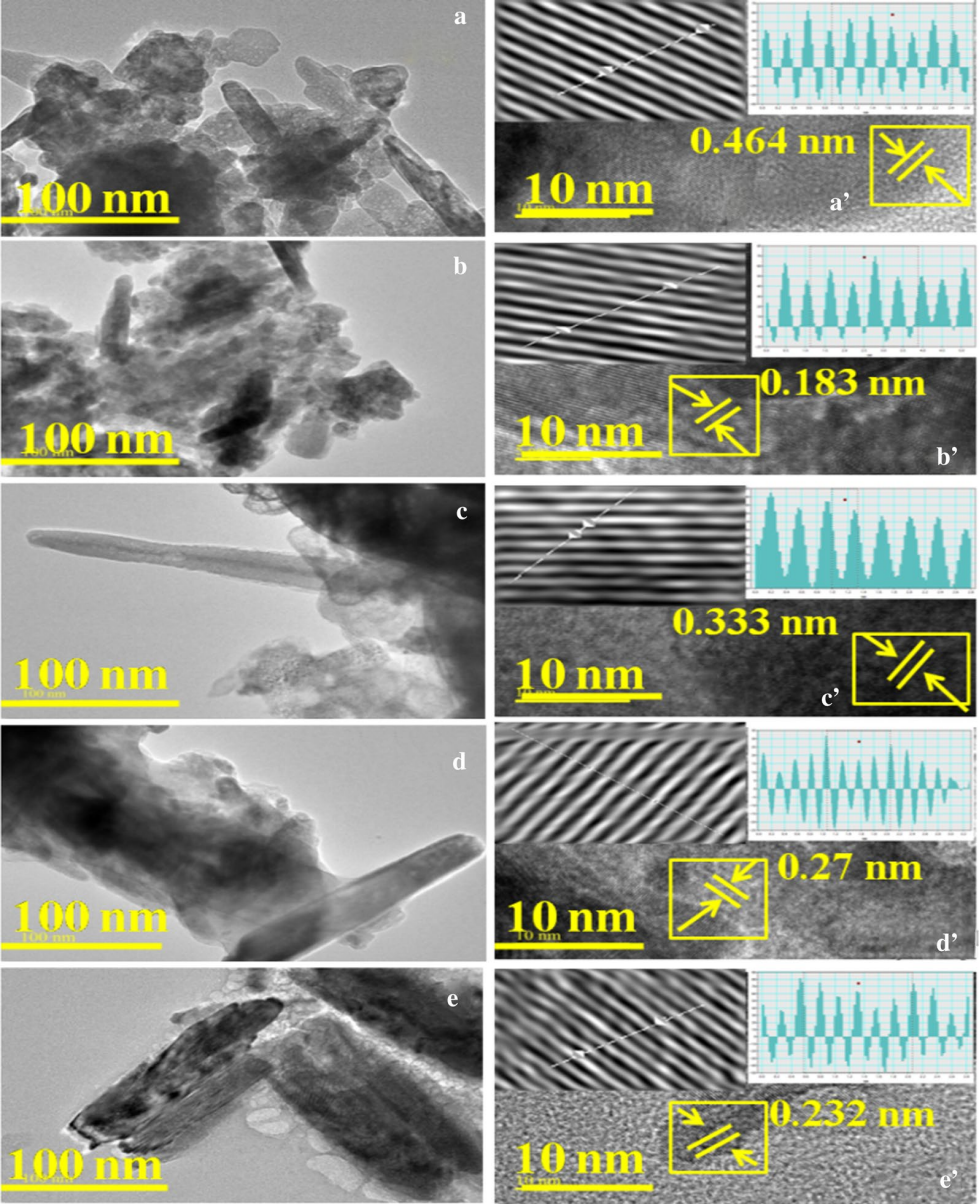
**a–e** HR-TEM images of various concentrations of Mg-doped ZnO and d-spacing calculated using HR-TEM images of Mg-ZnO **a**′**–e**′ with Mg content (2, 4, 6 and 8 wt%)

For morphological confirmation of undoped and doped ZnO, HR-TEM was carried out (Fig. 4a–e) to delineate hexagonal rod-like morphology of ZnO:Mg. It seems that Mg showed the role of nucleation as it grows with doping [39]. Interlayer d-spacing values for undoped and doped ZnO were calculated ~ 0.464, 0.183, 0.333, 0.27 and 0.232 nm HR-TEM images (Fig. 4a′–e′). The d-spacing values are well in agreement with planes obtained with XRD analysis. No presence of impurities/secondary phases suggest adequate incorporation of dopant atoms into ZnO nanorods without clustering [46]. Furthermore, change in d-spacing was attributed to Mg incorporation in ZnO lattices.

**Fig. 5:**
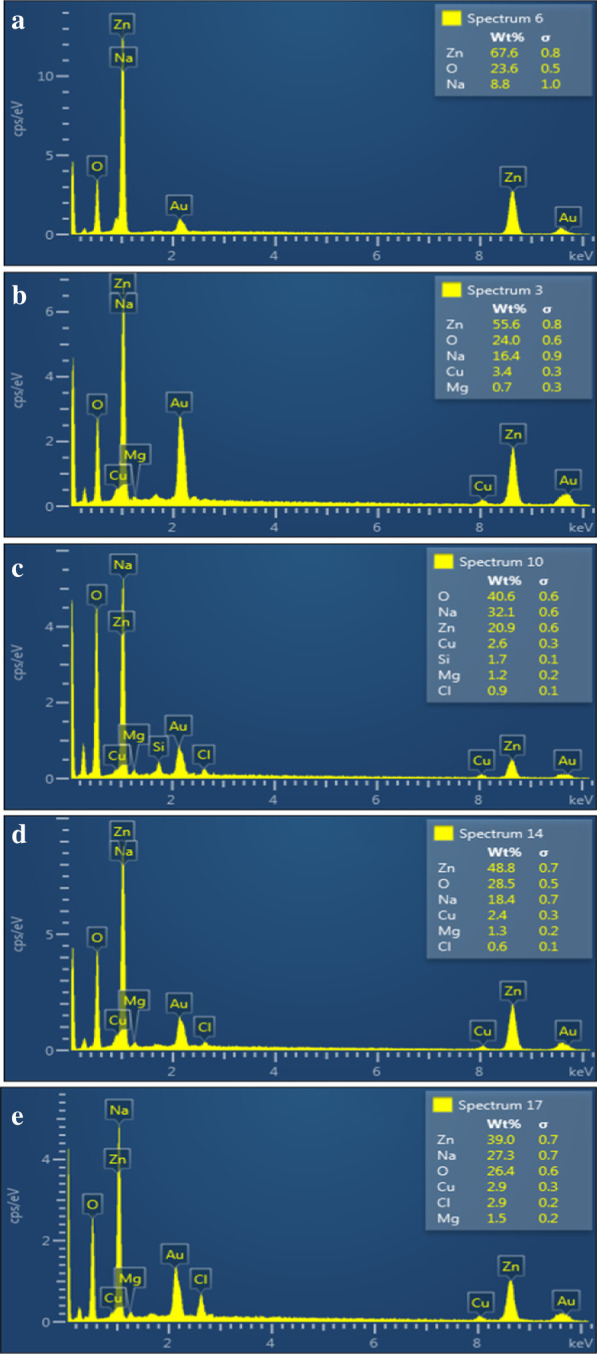
**a** EDS analysis of ZnO and various concentrations (2, 4, 6 and 8 wt%) of Mg-doped ZnO (**b–e**), respectively

Elemental analysis was performed using EDS to confirm the presence of zinc and oxygen in ZnO nanopowders (Fig. 5a–e). The average atomic ratio (67.6:23.6) quantitatively confirmed ZnO formation along with the dopant. Gold (Au) peaks appear in spectra due to the gold coating sputtered over the sample to reduce charging effect. Cu peaks may originate from the Cu tape used with the sample holder. Some additional peaks (Cl, Si) may indicate contamination. Na peak may have originated from NaOH that was used to maintain basic pH during synthesis. However, Na peak overlaps with Zn, so its presence in the sample cannot be ascertained.

**Fig. 6:**
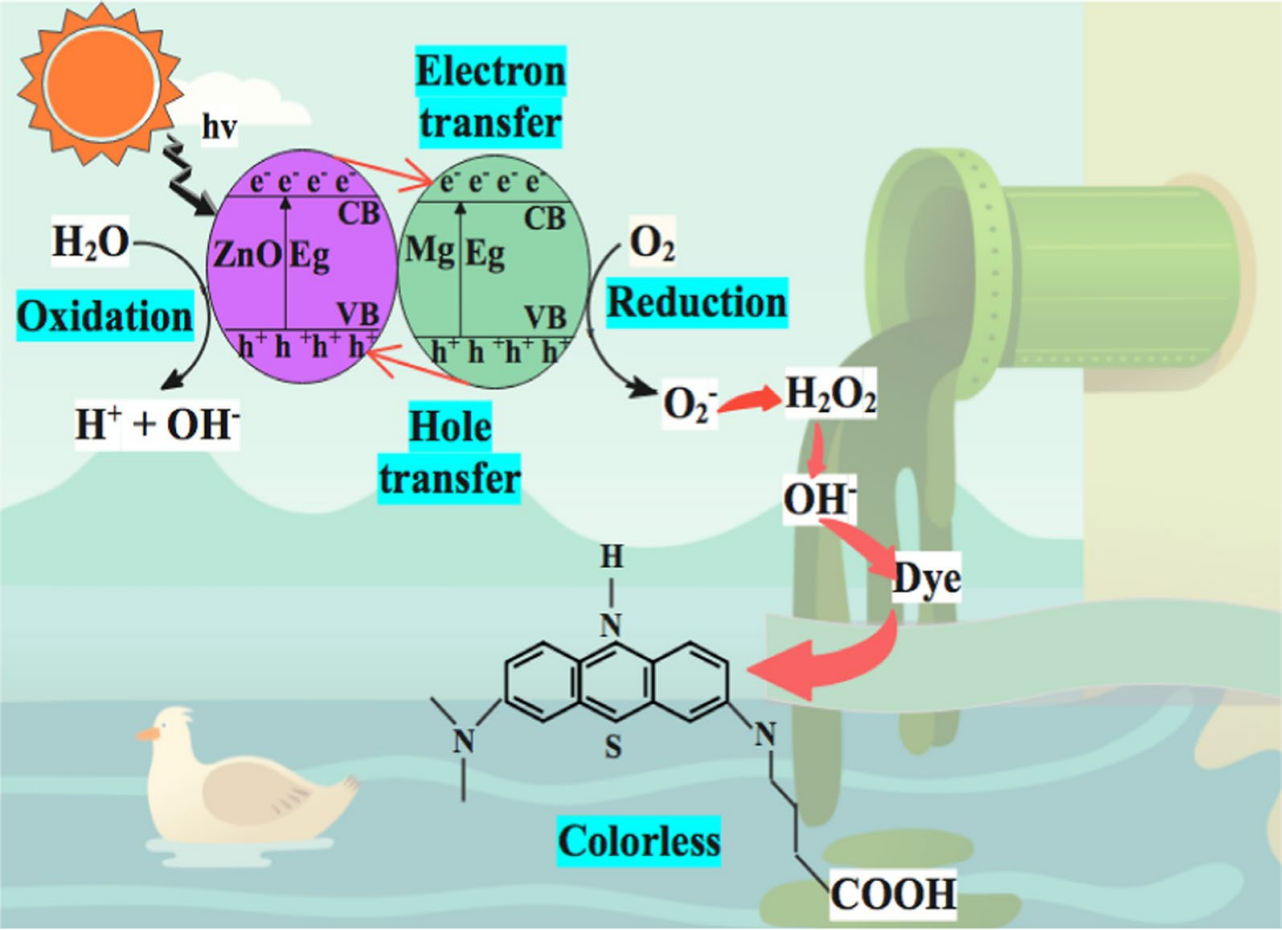
Schematic illustration of photocatalysis mechanism of Mg-doped ZnO nanorods

The photocatalytic process involves generation of electron–hole pairs (e-, h +) with succeeding separation and recombination of electrons and holes (Fig. 6), demonstrating the following redox reaction [35].

$$\begin{aligned} & {\text{ZnO}} + h\nu \to {\text{ZnO}}\,({\text{e}}_{{{\text{CB}}}} + {\text{h}}_{{{\text{VB}}}} ) \\ & {\text{e}}_{{{\text{CB}}}} + {\text{O}}_{2} \to {\text{O}}_{2}^{\cdot - } \\ & {\text{O}}_{2}^{\cdot - \,} + {\text{dye}}\,{\text{degraded}}\,{\text{products}} + {\text{CO}}_{2} + {\text{H}}_{2} {\text{O}} \\ & {\text{OH}}^{\cdot} + {\text{dye}}\,{\text{degraded}}\,{\text{products}} + {\text{CO}}_{2} + {\text{H}}_{2} {\text{O}} \\ \end{aligned}$$

**Fig. 7:**
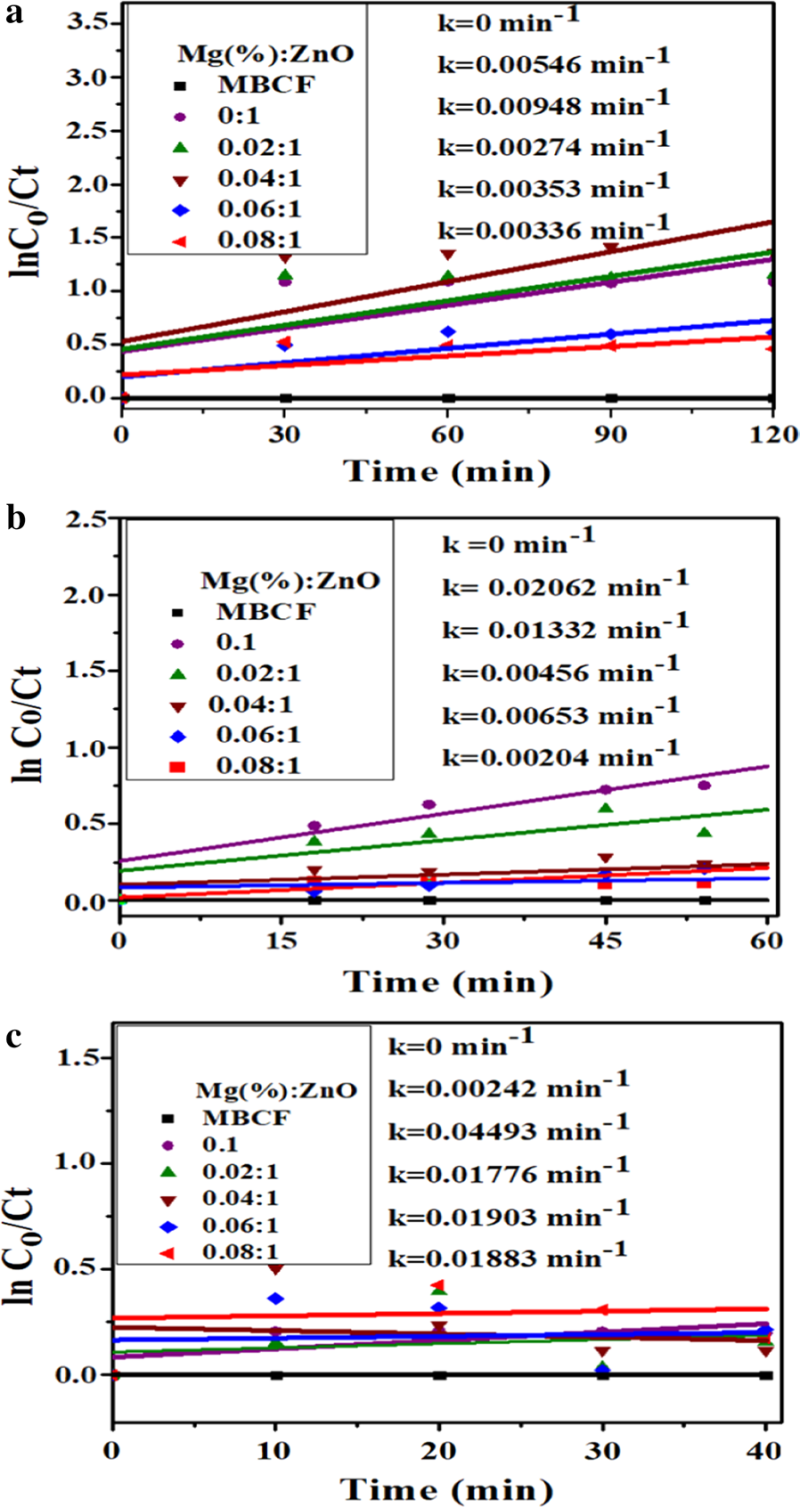
**a** Photocatalysis, **b** sonocatalysis and **c** sonophotocatalysis reaction kinetics of MBCF dye degradation for Mg-doped ZnO nanorods

All prepared samples were evaluated for their photocatalytic, sonocatalytic and sonophotocatalytic activities against MBCF as targeted contaminant. The degradation profiles of the MBCF dye-photocatalyzed under UV light irradiation by synthesized nanocatalysts are displayed in Fig. 7a–c.

**Fig. 8:**
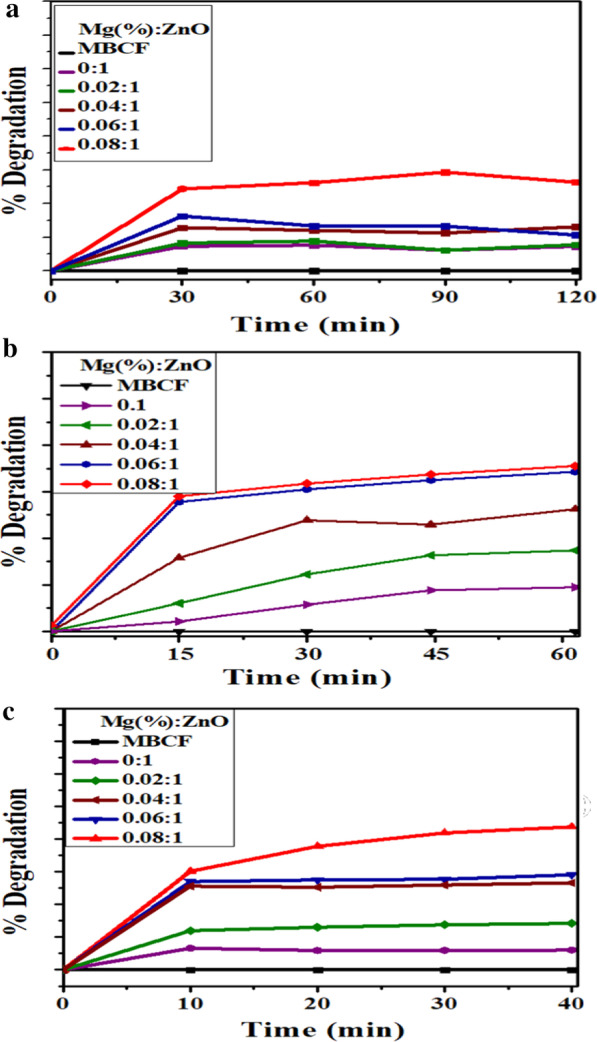
**a** Photocatalysis, **b** sonocatalysis and **c** sonophotocatalysis photodegradation of MBCF for Mg-doped ZnO nanorods

The pseudo-first-order kinetics-based k (rate constant) was determined by plotting linear curves of ln(Ct/Co), against exposure time t. Degradation rate constant k for undoped and doped ZnO (2, 4, 6 and 8 wt %) was calculated to be 0.00546, 0.00948, 0.00274, 0.00353 and 0.00336 min^−1^, respectively (Fig. 7a). Doped ZnO was found to have better photocatalytic efficiency than pure ZnO with a maximum degradation of 26% for doped ZnO (8 wt%) (Fig. 8a–c). Owing to the presence of surface oxygen vacancies, increased surface area was the explanation behind the increased photocatalytic activity of doped ZnO [35]. Photo-induced transfer of electrons in a semiconductor’s CB with positive holes left in VB is the fundamental mechanism of photocatalysis [15]. Until the excitons are annihilated, they take part in redox reactions with surrounding dye molecules on the surface of catalysts, resulting in degraded products. The photo-induced electrons act as strong reducing agent, which interact with surrounding O_2_ molecules to generate reactive O_2_^·−^ species. On the other hand, photo-induced holes act as strong oxidizing agent that generates highly reactive OH^·^ species from hydroxyl groups. The resultant radical species (O_2_^·−^ and OH^·^) interact with the surrounding dye molecules to degrade them into non-toxic products or minerals.

An alternate approach for efficient degradation of organic wastes in water is sonocatalysis (SC) [15]. The influence of ultrasonic waves on MBCF degradation was studied with undoped and doped ZnO. (Fig. 7b). In terms of MBCF dye concentration, SC degradation of MBCF by doped ZnO followed pseudo-first-order kinetics. Degradation rate constants of undoped and doped ZnO (2, 4, 6 and 8 wt%) were 0.02062, 0.01332, 0.00456, 0.00653 and 0.00204 min^−1^, respectively. Several studies were recently reported on SC dye degradation, based on hot-spot mechanism and sonoluminescence, in the presence of various catalysts. Cavitation bubbles’ formation in solution can be boosted by creating hot spots through asymmetric nucleation of bubbles. These hot spots may trigger OH to be formed by H_2_O molecules to pyrolyze. Sonochemical mechanism typically requires water sonolysis, which is the solvent under high pressure and temperature within the collapsing cavitation bubbles. In MBCF and nanocatalyst solution, ultrasonic waves not only cause water sonolysis, but also catalyst couple to create charge carriers. OH radicals and superoxide anions ^·^O^2−^ can be generated by electron–hole pairs, which decompose dyes into non-toxic species [15, 47]. Sonophotocatalysis (SPC) also appears to follow pseudo-first-order kinetics, similar to photocatalysis and sonocatalysis. Degradation rate constants for undoped and doped ZnO (2, 4, 6 and 8 wt %) were 0.00242, 0.04493, 0.1776, 0.01903 and 0.01883 min^−1^, respectively (Fig. 7c). Degradation performance of doped ZnO was 12, 29, 53, 58 and 87%, respectively (Fig. 8c).

**Fig. 9:**
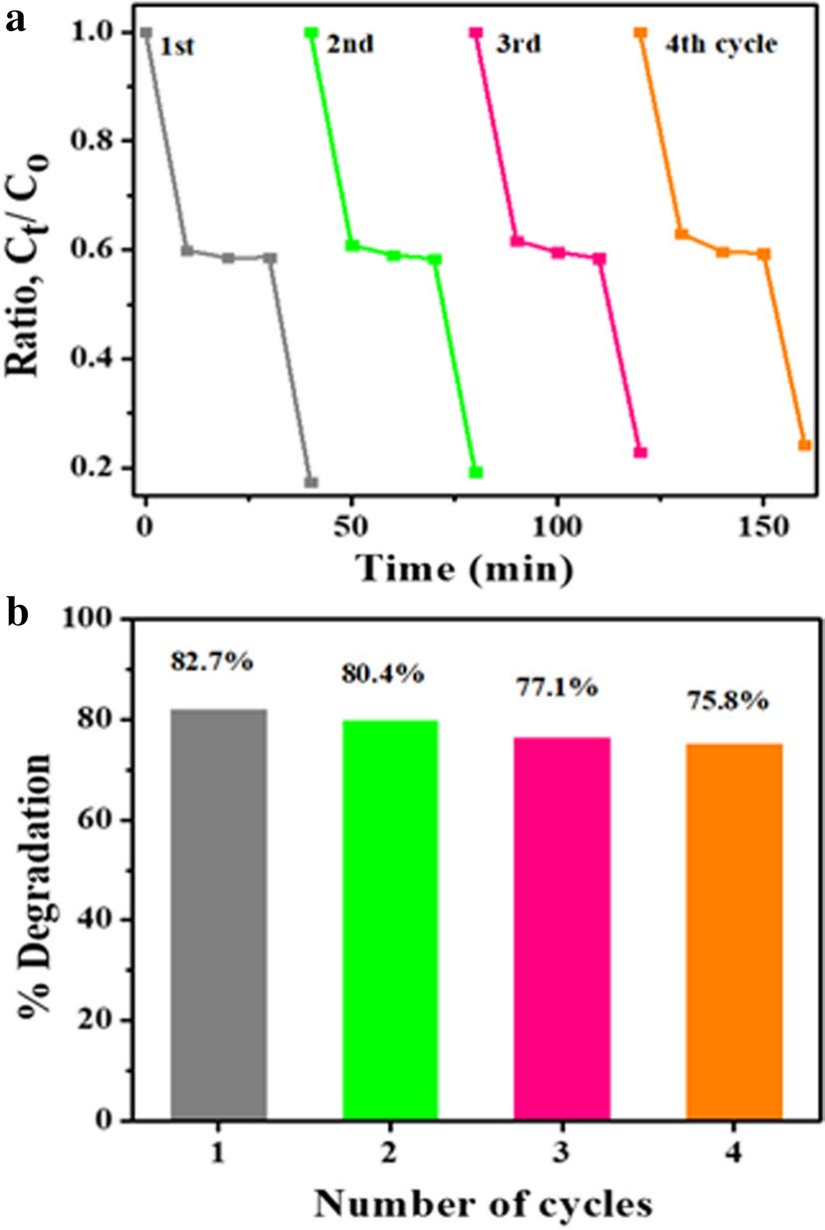
**a** Photocatalysis reusability performance of Mg-doped ZnO and **b** %degradation bar graph

These results suggest that doping plays a crucial role in the efficiency of ZnO photocatalytic. At identical operating conditions, SPC has a higher degradation rate than the corresponding individual mechanisms. The combined process reaction rate constant is greater than the sum of individual processes' rate constants, i.e., photo of Ksono > Kphoto + Ksono, which can be attributed to (i) increase in OH production in mixture, (ii) raised transfer of mass between solution and catalyst surface, and (iii) enhanced activity related to ultrasound disaggregation, consequently enhancing the area of surface [15, 48]. In order to estimate the reusability as well as sample steadiness, Fig. 9a indicates that photocatalytic switches off MBCF colorant degradation under similar conditions after back to back (four cycling experiments). In this way, sample’s degradation efficiency reduced from to 82 to 75%. Herein (Fig. 9b), there is some depletion of nanomaterial by centrifugation or washing while doing recycling experiment. Following the recycling results, it was concluded that the product lasts stable and possesses remarkable ability and acceptance for dangerous wastewater treatment. Anyhow, Table 2 shows the comparison of photocatalytic degradation efficiency of present work with other reported materials.

In vitro bactericidal action of undoped and doped ZnO NRs for G-ve and G+ve bacteria is given in Table 1. Results depict improved bactericidal synergism and action of doped ZnO against *E. coli* in contrast to *S. aureus*. Inhibition zones were recorded as (1.05–2.05 mm) and (2.10–4.15 mm) for *S. aureus* and (0–6.15 mm) to (0–8.65 mm) for *E. coli*, respectively, while ZnO showed negligible efficacy for *E. coli* as compared to *S. aureus*. Moreover, control + ve depicted inhibition zone (9.00 mm) against *E. coli* and *S. aureus* parallel to control -ve (0 mm). Overall, Mg-doped ZnO exhibited substantial (P < 0.05) efficacy against G-ve as compared to G+ve bacteria.

**Table 1:** Antibacterial activity of Mg-doped ZnO nanorods

Sample	Inhibition zone^a^ (mm)		Inhibition zone^b^ (mm)	
	0.5 mg/50 μl	1.0 mg/50 μl	0.5 mg/50 μl	1.0 mg/50 μl
ZnO	1.05	2.10	0	0
Mg (0.02):ZnO	1.25	2.55	0	3.90
Mg (0.04):ZnO	1.45	2.90	5.40	7.15
Mg (0.06):ZnO	1.60	3.45	5.85	7.50
Mg (0.08):ZnO	2.05	4.15	6.15	8.65
Ciprofloxacin	9.00	9.00	9.00	9.00
DI water	0	0	0	0

^a^Inhibition zone diameters (mm) for *S. aureus*

^b^Values of zones of inhibition for *E. coli*

**Fig. 10:**
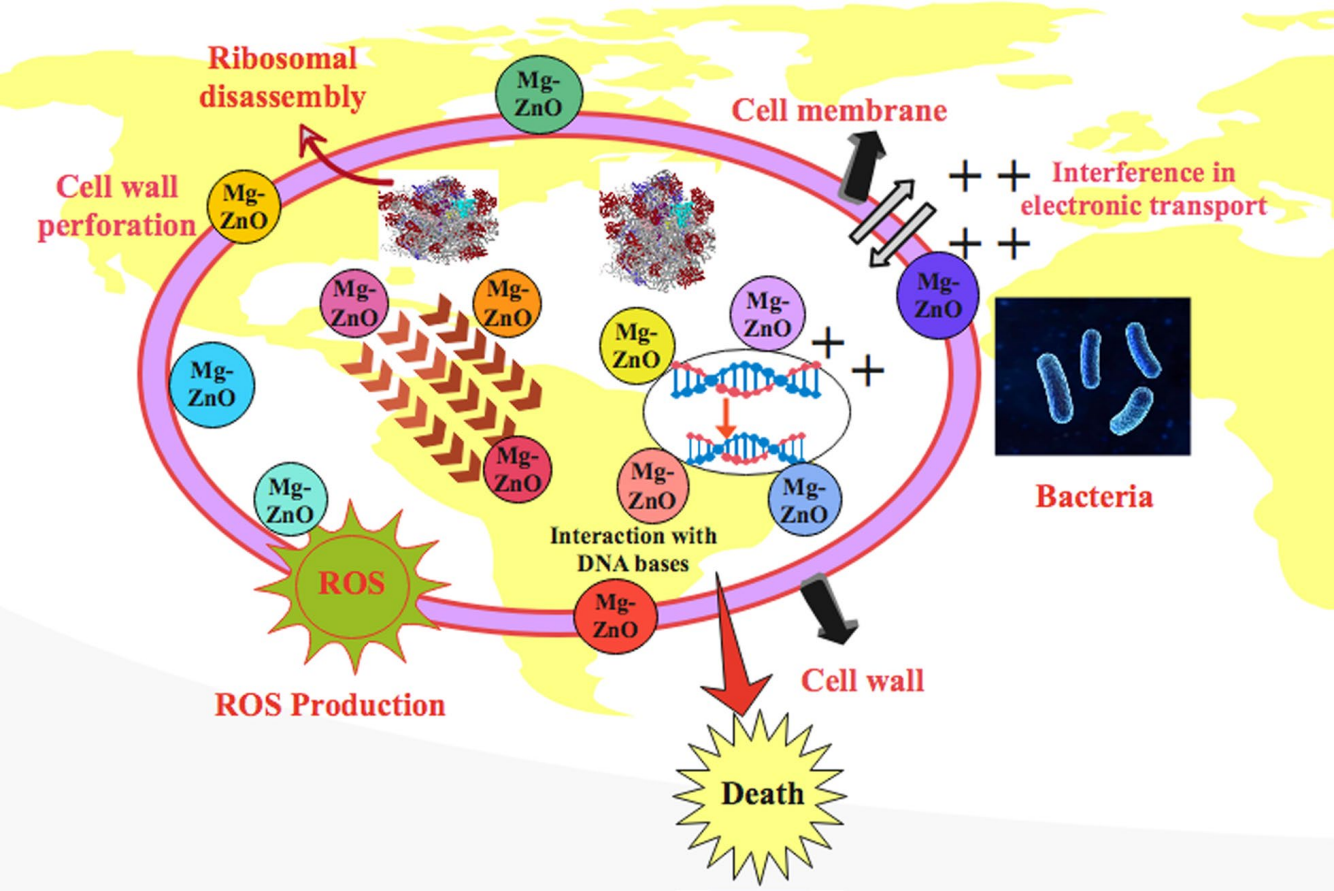
Schematic illustration of bactericidal mechanism of Mg-doped ZnO nanorods

Oxidative stress induced by prepared doped ZnO depends upon concentration, shape and size of NRs, while increment in NRs size reduces antibacterial activity. Nanosized rods generate oxygen species (ROS) to produce bacterial cell membrane as a result of extrusion of cytoplasmic content, which cause bacteria death as shown in Fig. 10. Another possible phenomenon involves strong interaction between negatively charged cell membrane and cations (Mg^2+^ and Zn^2+^) that results in crumbling of micro-pathogens [49].

Drug resistance has been considered as major threat to mankind, and there is continuous need for discovery of more compatible antibiotics. Bactericidal activity of metal NRs is well documented, and their role as possible candidate for new antibiotic discovery has been suggested previously [50]. In silico molecular docking studies facilitate to get insight into mechanism behind their antibacterial activity. Dihydrofolate reductase (DHFR) and dihydropteroate synthase (DHPS) enzyme belonging to folate biosynthetic pathway have been reported as well-known target for trimethoprim and sulfonamide drugs, respectively [51, 52]. Here, we evaluated binding tendency of Mg-doped ZnO NRs against DHFR, DHPS and FabH enzymes from *E. coli*. Docked complexes revealed their binding pattern inside active site and suggested them as possible inhibitor against selected enzyme targets.

**Fig. 11:**
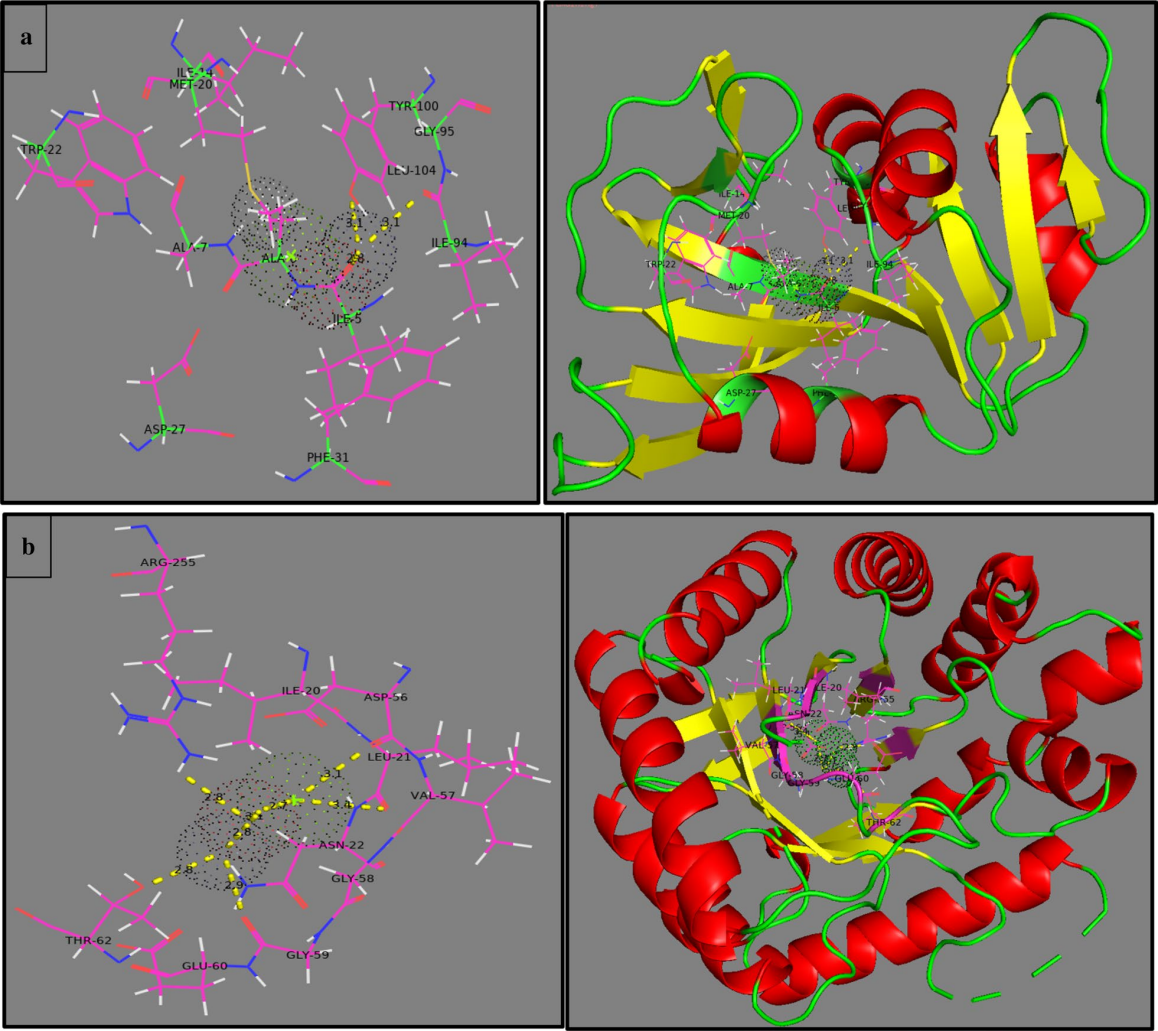
Binding interaction pattern of Mg-doped ZnO NRs inside active pocket **a** Dihydrofolate reductase (DHFR), **b** Dihydropteroate synthase (DHPS) from *E. coli*

For DHFR_E.coli_, the best docked complex revealed H-bonding interaction with Ile94 (3.1 Å), Tyr100 (3.1 Å) and metal–contact interaction with Met20 and Ala7 with overall binding score -7.518 kcal/mol. Binding interactions with key amino acids of active pocket and orientation of Mg-doped ZnO NP are depicted in Fig. 11a.

For DHPS_E.coli_, docking complexes showed H-bonding with Leu21 (3.1 Å), Asp56 (3.4 Å), Gly59 (2.9 Å), Thr62 (2.8 Å) and Arg255 (2.8 Å). In addition, the Asn22 and Ile20 interacted with NRs through metal contact inside active site as shown in Fig. 11b. These Mg-doped ZnO NPs blocked active site (binding score: -6.973 kcal/mol) and are suggested to be possible inhibitors against DHPS enzyme.

**Fig. 12:**
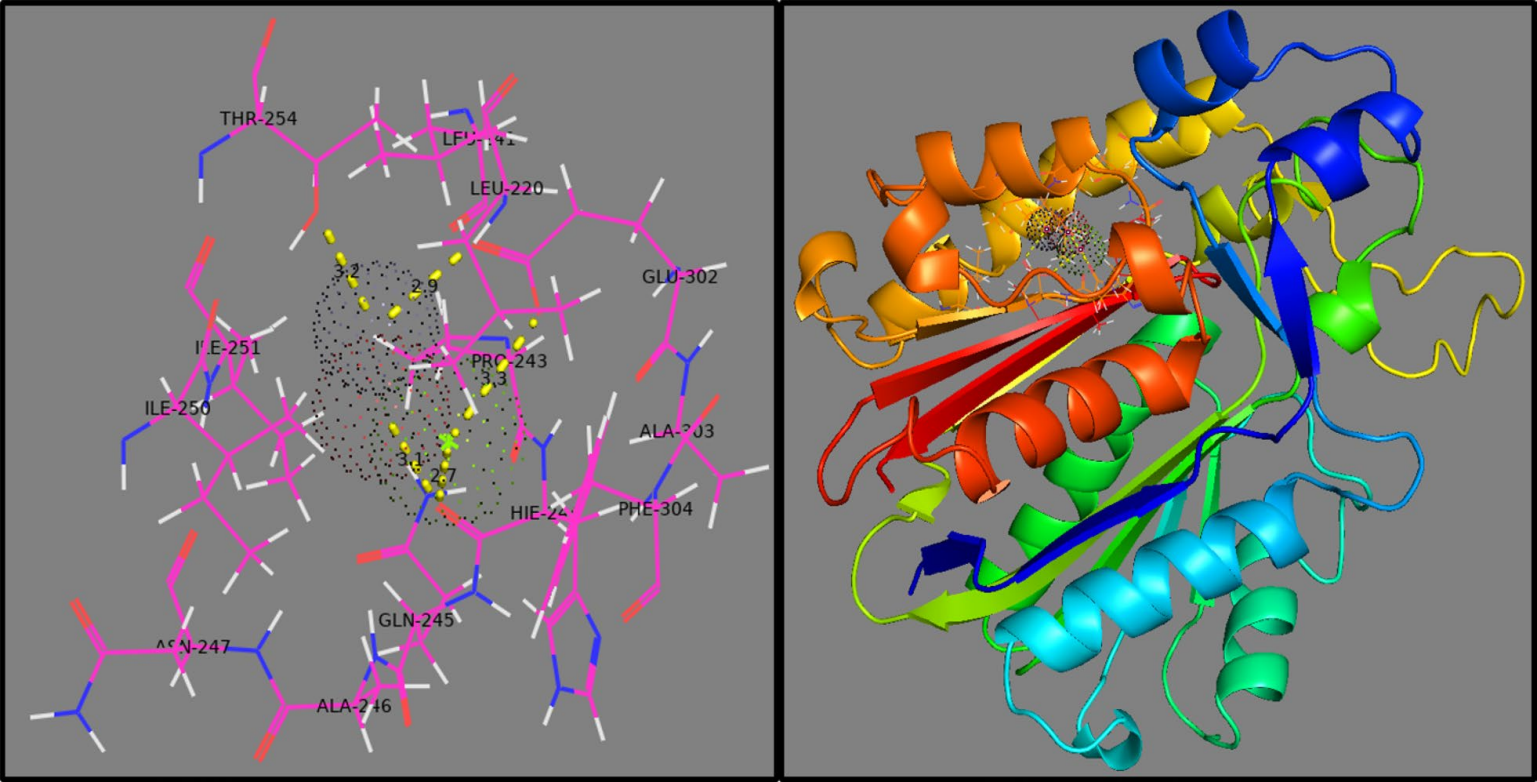
Binding interaction pattern of Mg-doped ZnO NRs inside active pocket *β*-ketoacyl-acyl carrier protein synthase III (FabH) from *E. coli*

Similarly, docking of Mg-doped ZnO NRs against the *β*-ketoacyl-acyl carrier protein synthase III (FabH) enzyme of fatty acid biosynthetic pathway showed H-bonding interaction with Glu302 (3.3 Å), Leu220 (2.9 Å), Thr254 (3.2 Å), and Gln245 (2.7 Å) having binding score -6.548 kcal/mol (Fig. 12). Furthermore, Mg-doped ZnO NPs involved metal contact interaction with Ile250 and His241.

Blockage of active site through binding of ligands prevents entry of substrate and thus leads to loss of enzyme activity. Owing to better antibacterial activity of Mg-doped ZnO NRs against *E. coli* as compared to *S. aureus*, in silico predictions against selected enzyme targets revealed their possible binding patterns inside active pocket and suggested them potential inhibitors of given enzymes.

A comparison of present sonophotocatalytic study with the literature is shown in Table 2.

**Table 2:** Comparison table of photocatalysis efficiency of Mg-doped ZnO nanorods with other reported materials

Material	Dye	Catalyst amount	Time (min)	Degradation result (%)	References
CuO nanosheets	Congo Red	0.05 g in 100 ml of CR	210	12	[53]
ZnO/ZnS core shell NPs	Rose Bengal	0.05 g in 100 ml of RB	120	50	[54]
CdS/ZnO	Methylene blue	0.02 g in 20 ml of MB	300	71	[55]
ZnO NPs	Congo Red	0.05 g in 100 ml of CR	120	85	[56]
ZnO/Ag_2_S core shell NPs	Eriochrome Black T (EBT)	0.05 g in 100 ml of EBT	120	69	[57]
Fe-doped ZnO NPs	Methylene orange	0.02 g in 100 ml of MO	120	72	[58]
Mg-doped ZnO NRs	Methylene Blue Ciprofloxacin (MBCF)	10 mg in 50 ml of MBCF	40	87	Present work

## Conclusion

Using co-precipitation technique, Mg-doped ZnO NRs were successfully synthesized, and the influence of Mg doping on the phase constitution, elemental composition, morphology and optical properties of ZnO was investigated. Using XRD analysis, the ZnO has hexagonal wurtzite phase, while the estimated crystallite size was less than 100 nm. Crystalline structure of ZnO was also improved by Mg doping, which in turn led to increased luminescence and an increase in the band gap. UV–Vis absorption spectra revealed blueshift indicating band gap widening, while ZnO rod formation was confirmed by EDS study, where an average atomic ratio of 67.6:23.6 was observed. Raman spectrum was blueshifted for higher values of doping (8 wt%) caused by substitution of Mg^2+^ for Zn^2+^ in ZnO lattice. PL results indicated increased visible emissions with Mg, leading to an increase in electron hole pair delocalization. Dye degradation performance of synthesized NRs was evaluated against MBCF, and best results were obtained via sonophotocatalysis with maximum degradation efficiency of 87% for Mg-doped ZnO. Inhibition zones were recorded as (1.05–2.05 mm) and (2.10–4.15 mm) for *S. aureus* and (0–6.15 mm) to (0–8.65 mm) for *E. coli*, respectively. Therefore, doped nanorods may be imposed as a control material to minimize antibiotic resistance. Furthermore, in silico molecular docking studies predicted Mg-doped ZnO NRs as potential inhibitor of DHFR, DHPS and FabH enzyme. The inhibition of given enzymes is suggested as possible mechanism behind bactericidal activity of Mg-doped ZnO NRs against *E. coli*.

## Data Availability

All data are fully available without restriction.

## Abbreviations

DHFR: Dihydrofolate reductase
DHPS: Dihydropteroate synthase
EDS: Energy-dispersive X-ray spectroscopy
FTIR: Fourier transform infrared spectroscopy
FESEM: Field emission scanning electron microscopy
G+ve: Gram-positive
G-ve: Gram-negative
HR-TEM: High-resolution transmission electron microscopy
JCPDS: Joint Committee on Powder Diffraction Standards
Mg: Magnesium
UV–Vis: Ultraviolet–visible spectroscopy
XRD: X-ray diffraction
ZnO: Zinc oxide
